# Autoimmune polyglandular syndrome type 2 in an HIV-positive man managed at the University Teaching Hospitals Lusaka, Zambia: a case report

**DOI:** 10.11604/pamj.2022.41.31.30195

**Published:** 2022-01-12

**Authors:** Mwila Meek, Chishiba Kabengele, Brown Kamanga

**Affiliations:** 1 The University of Zambia School of Medicine, Lusaka, Zambia,; 2 Young Emerging Scientists (YES), Lusaka, Zambia,; 3 Rwanda Zambia Health Research Group (RZHRG), Emory University, Atlanta, GA, USA,; 4 University Teaching Hospitals, Department Of Medicine, Endocrine Unit, Lusaka, Zambia

**Keywords:** Graves’ disease, type 1 diabetes mellitus, case report

## Abstract

Autoimmune polyglandular syndromes (APS) are rare autoimmune endocrinopathies characterized by the coexistence of at least two endocrine gland insufficiencies developed from autoimmune mechanisms. APS may also be associated with non-endocrine immune diseases. In HIV infection, antiretroviral therapy can improve the quality of life to reduce the incidence of opportunistic infections, malignancies, and death. HIV disease may also be associated with complications, such as immune reconstitution inflammatory syndrome (IRIS) presenting as infections, malignancies or autoimmune diseases. We here report the clinical case of an HIV-infected man receiving antiretroviral therapy, who subsequently developed APS type II, characterized by Grave’s disease, type 1 diabetes mellitus. He complained of a mass in his anterior neck, diarrhea, weight loss, palpitations, hand tremors and excessive sweating. Six months before he had been diagnosed with type 1 diabetes mellitus. The patient had a diffusely enlarged thyroid on ultrasound, elevated random blood glucose of 14.0 mmol/l; elevated free T4 at 5.03 ng/dL and suppressed thyroid-stimulating hormone (TSH) at <0.05 micro-IU/mL. The patient was treated with carbimazole and propranolol for Graves’ thyrotoxicosis and basal bolus insulin regimen (actrapid and protaphane) for hyperglycemia. At monthly follow-up assessments he was euthyroid and 2-hour postprandial blood glucose test was normal. The goitre had markedly reduced in size. This screening for APS in HIV patients with autoimmune IRIS as well as patients with autoimmune endocrinopathies in order to allow for early diagnosis and prompt initiation of treatment to reduce the risk of morbidity and mortality.

## Introduction

Autoimmune polyglandular syndromes (APS) are multifactorial diseases with at least two coexisting autoimmune-mediated endocrinopathies. Apart from autoimmune endocrinopathies, APS brings together neurologic, dermatologic, gastrointestinal and other disorders which share an autoimmune pathogenesis [1]. In most endocrinopathies that constitute APS, the autoimmune process causes an irreversible loss of the affected gland function of the [2].

Two significant subtypes of Autoimmune polyglandular syndrome have been described; Autoimmune polyglandular syndrome type I (APS-I) and Autoimmune polyglandular syndrome type II (APS-II). The two differ in terms of onset, gender distribution, transmission modality, and the presence of various autoimmune diseases. A third type called Autoimmune polyglandular syndrome type III (APS-III), which occurs in adults, though rarely, has been described. APS-III includes two of the following: thyroid deficiency, pernicious anaemia, type 1 diabetes mellitus (T1DM), vitiligo and alopecia and does not involve the Adrenal cortex [3].

Autoimmune polyglandular syndrome type I, also known as Whitaker syndrome, is a disease with an autosomal recessive inheritance. It results from mutations in the autoimmune regulator (AIRE) gene leading to the loss of central tolerance, a process by which developing T cells with potential reactivity against self-antigens are eliminated during early differentiation in the thymus [4]. In patients, the first symptoms of APS-I usually appear in childhood, with complete picture occurring in their twenties. APS-I is associated with mucocutaneous candidiasis, hypoparathyroidism, and Addison´s disease (autoimmune primary adrenal failure). However, cases of APS-I without mucocutaneous candidiasis have been reported [4,5].

Autoimmune polyglandular syndrome type II is the most common type of autoimmune polyglandular syndromes. Addison´s disease characterizes it in combination with autoimmune thyroid disease and/or T1DM. Primary hypogonadism, myasthenia gravis and celiac disease have been also commonly observed in this syndrome. However, cases of APS-II without Adrenal gland involvement have also been reported [6]. Autoimmune polyglandular syndrome type II is associated with HLA-DR3 and/or HLA-DR4 haplotypes with a variably expressed autosomal dominant inheritance pattern. The diagnosis of APS-II is usually made in patients between 20 and 60 years, with middle-aged women having a more increased prevalence than men. We report a case of autoimmune polyglandular syndrome type 2 in an HIV-positive man managed at the University Teaching Hospitals in Lusaka, Zambia.

## Patient and observation

A 27-year-old male presented to the Department of Emergency at the University Teaching Hospitals´ (UTH) complaining of a neck mass, diarrhoea, and weight loss. He also had palpitations, hand tremors and excessive sweating.

Six months before, he was admitted to the emergency ward (EW) of the UTH in Lusaka on referral from the local clinic where he had presented with a three-week history of increased urinary frequency, thirst, and appetite. He had a history of HIV and smear-positive pulmonary tuberculosis diagnosed ten and five years ago, respectively. About five years ago, he had received second-line combination antiretroviral therapy ((cART), i.e., Abacavir, Lamivudine, Lopinavir/ritonavir), following treatment failure of a first-line regimen (Tenofovir, Lamivudine, Efavirenz) because of poor adherence to treatment. His paternal uncle also had diabetes mellitus. The patient was diagnosed with type 1 diabetes mellitus based on high random blood glucose, HbA1c, Anti-GAD and IA2 levels. The remainder of the lab tests performed on his first admission are summarized in Table 1. On admission, the patient received subcutaneous basal-bolus insulin (Actrapid and Protaphane), a reduction in blood glucose to target levels. He was discharged five days later on insulin, and he continued his cART.

**Table 1:** summary of laboratory tests done during the patient’s first admission

Test Done	Findings	Reference Range	Flag
Urinalysis			
Specific gravity	1.015	1.010-1.020	
Ketones	Negative		
Glucose	Negative		
Casts	Negative		
Haemoglobin concentration	14.3g/dL	12-17	
Hb1Ac	7.2%	4-6.5	High
Electrolytes (mmol/L)			
Potassium	4.2	3.5-5.0	
Calcium (corrected)	2.3	2.0-2.6	
Sodium	136	135-145	
Random serum glucose (mmol/L)	15	7-11	High

**Figure 1:**
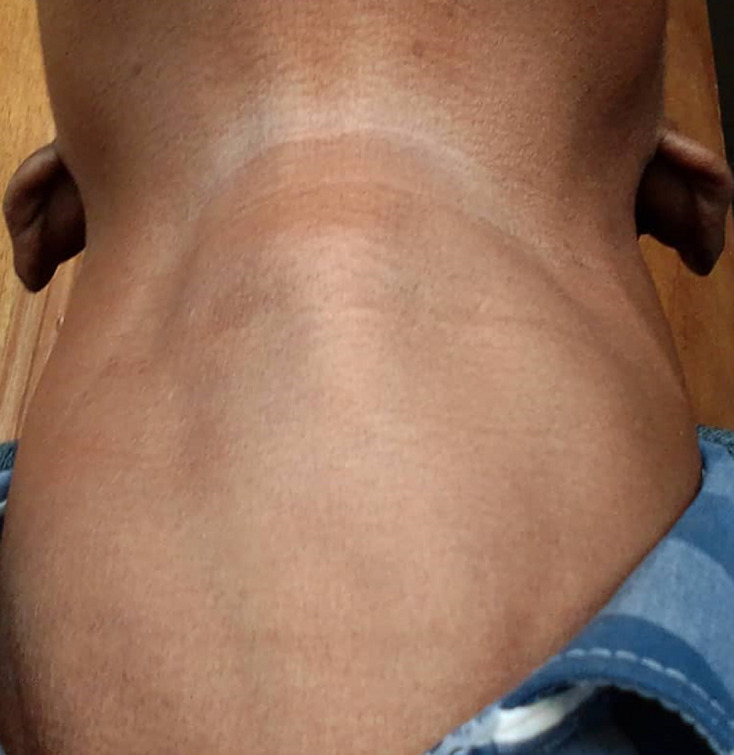
the patient’s anterior neck

**Clinical findings:** physical examination revealed a low-normal body mass index of 18.2 kg/m^2^ (weight 52Kg, height 169cm), mild conjunctival pallor, low volume pulse, and an increased regular resting heart rate of 101 beats/minute, synchronic with pulse rate, normal blood pressure without postural drop (laying BP 124/75mmHg; standing BP 120/75mmHg). He had a non-tender, bi-lobed mass in the anterior neck below the laryngeal prominence (Figure 1), moving with swallowing. The left and right lobes measured 4.0 x3.0 cm and 4.8x3 cm, respectively. A bruit was heard over the mass on auscultation. His hands were tremulous. He had a weak grip and could not raise his arms or stand up against resistance, suggesting proximal muscle weakness. Both his finger and toenails were clubbed. His skin was warm and sticky. There was no vitiligo, cervical lymph node enlargement or neck vein engorgement. He did not have pre-tibial myxedema, thyroid eye changes, or breast enlargement. His respiratory and gastrointestinal examinations were also unremarkable.

**Diagnostic assessment:** laboratory results revealed high random blood sugar level of 14.0 mmol/l; high free T4 at 5.03 ng/dL (reference range: 0.89-1.76 ng/dL) and suppressed thyroid-stimulating hormone (TSH) at <0.05 micro-IU/mL (reference range: 0.25-4.50 micro IU/mL). He had slightly elevated tissue transglutaminase IgA and IgG at 0.60 IU/mL (reference: <0.7 IU/mL), and small bowel biopsy was performed to confirm celiac disease. Results were reported as negative for parietal antibodies and intrinsic factor antibodies. Vitamin B12 levels were normal (274 pmol/L [reference: 138 pmol/L], and so were urine tests (Table 2). Thyroid ultrasonography revealed diffusely enlarged thyroid gland with smooth margins, reduced echogenicity, and increased vascularity. Left lobe measured approximately 4.97cm x 2.56cm x 3.89cm with a volume of 24.36ml. Right lobe measured about 5.01cm x 2.52cm x 3.23cm with a volume of 21.38ml. No neck lymph node enlargement, areas of calcification, nodules or cystic lesions were noted. Based on these results, the diagnosis of APS II was made.

**Table 2:** patient’s laboratory results

Test Done	Findings	Reference Range	Flag
Thyroid Function Test			
TSH	< 0.05	0.25- 4.50	Suppressed
	microIU/ml	microIU/ml	
Free T4	5.03ng/dl	0.89-1.76ng/dl	Elevated
Tisssue	0.7 IU/ml	< 0.7IU/ml	Slightly
transglutaminase			Raised
IgA and IgG			
Serum Vitamin B12	274pmol/L	>138pmol/L	Normal
Parietal cell antibodies Intrinsic factor	Negative		Negative
antibody			
TSH receptor antibody	22.87 IU/L	<1.80 IU/L	Positive
ANTI-GAD antibody	>2000 IU/Ml	<10 IU/L	Positive

**Therapeutic intervention:** the patient received carbimazole 40mg once daily, propranolol, twice daily, and - and subcutaneous insulin Actrapid 4 units daily, at 6-hour intervals, first dose at 06: 00hrs as well as Protaphane 10 units at 22: 00 hrs. The patient was transferred from basal bolus insulin to Actraphane at a dose of 14 units given 30 minutes before Breakfast and 8 units 30 minutes before dinner, subcutaneously as his blood glucose remained on targets. The patient did not consent to receive radioactive iodine 131 (RAI-131) therapy for thyroid ablation. Patient´s general condition improved and post-discharge follow-up program was started at the endocrine clinic.

**Follow up and outcome:** the patient was reviewed a month after discharge from the hospital. At the time of review, he was euthyroid, and 2-hour postprandial blood glucose test was still normal. The goitre had markedly reduced in size.

**Patient´s perspective:** during and after hospitalization, the patient was delighted with the care he received and was optimistic about the outcome of his condition.

**Informed concent:** the patient was informed about the case report, why his case was unique, and the authors being interested in publishing his case. He willingly gave informed consent to allow the authors to use his photos for this case report.

**Funding:** this work was supported through the Sub-Saharan African Network for TB/HIV Research Excellence (SANTHE), a DELTAS Africa Initiative [grant # DEL-15-006]. The DELTAS Africa Initiative is an independent funding scheme of the African Academy of Sciences (AAS)´s Alliance for Accelerating Excellence in Science in Africa (AESA) and supported by the New Partnership for Africa´s Development Planning and Coordinating Agency (NEPAD Agency) with funding from the Wellcome Trust [grant # 107752/Z/15/Z] and the UK government. The views expressed in this publication are those of the author(s) and not necessarily those of AAS, NEPAD Agency, Wellcome Trust or the UK government.

## Discussion

HIV positive individuals with weakened immune system (decreased CD4+ count) may develop opportunistic infections or autoimmune disease after immunity improves following combined antiretroviral therapy (cART). This phenomenon is called Immune Reconstitution Inflammatory Syndrome (IRIS) [6]. In Unmasking IRIS, the infection is newly identified after initiation of cART. Paradoxical IRIS refers to a previously treated infection that clinically worsens after initiation of cART. Autoimmune IRIS results from a robust immune response directed against autoantigens, which leads to autoimmune conditions involving both the endocrine and nonendocrine organs. Individuals with autoimmune diseases secondary to immune reconstitution may have a genetic predisposition to them [7]. Autoimmune thyroid disease is the most common immune reconstitution disease in patients receiving cART [7]. Very little information have been published on autoimmune diabetes mellitus resulting from autoimmune IRIS [7]. Autoimmune diseases in HIV infected individuals may also develop due to molecular mimicry in the acute phase of HIV infection.

Some authors have reported autoimmune IRIS in patients who had been on cART for 3 to 38 months [7]. In our patient, T1DM and GD were diagnosed after a three-year course (2016-2019) of second-line c-ART (Abacavir, Lamivudine, Lopinavir/ritonavir). Since initiation of second-line cART regimen, patient´s viral load was undetectable, and CD4+ count increased to 950 cells/mm^3^, suggesting adherence to treatment and improved immune status. Therefore, autoimmune IRIS was considered the mechanism for developing T1DM and Grave´s disease. Autoimmune T1DM is a T-cell mediated autoimmune disease, resulting in the destruction of insulin-producing pancreatic beta-cells of genetically susceptible individuals, and triggered by an environmental factor. Individuals with T1DM may also have other autoimmune conditions, such as Hashimoto´s thyroiditis, GD, Addison´s disease, Celiac disease, and Pernicious anaemia. Several lines of evidence indicate that pancreatic beta-cell destruction results from lymphocytes (commonly T-cell) and macrophages infiltration in pancreatic islets and the presence of autoantibodies to islet cell antigens (ICA) tyrosine phosphatase (IA2A), glutamic acid decarboxylase (GADA) and insulin (IAA). Glutamic acid decarboxylase antibodies last longer and patients have been shown susceptibility to develop general autoimmunity. Anti-IA2 is a more specific marker of beta-cell destruction [7]. Glutamic acid decarboxylase and IA2 antibodies were positive in our patient, suggesting TIDM.

Grave´s hyperthyroidism is caused by TSH receptor stimulating antibodies, which bind to and activate the TSH receptor on thyroid cells. These stimulatory TSH receptor antibodies cause thyroid hormone hypersecretion and have an anabolic effect on thyroid follicular cells, resulting in thyroid follicular cell hypertrophy and hyperplasia. Thyroid cell hypertrophy and hyperplasia consequently led to goitre formation and increased thyroid hormone production. Grave´s disease affects approximately 0.5% of general population and is the commonest cause of hyperthyroidism worldwide (responsible for 50-80% of cases of hyperthyroidism) [8,9]. Thyroid ultrasound in patients with GD show diffusely enlarged thyroid with increased vascularity. In our patient thyroid ultrasound revealed these changes.

In patients with Graves´ hyperthyroidism, thyroid function tests reveal suppressed TSH levels, increased serum levels of free T3 and free T4, as well as positive TSH receptor antibodies. In our patient, thyroid function tests showed TSH suppression and elevated free T4. TSH receptor antibody was positive, confirming GD hyperthyroidism. Other autoimmune conditions such as pernicious anaemia and celiac disease were excluded. Apart from low-normal BMI, mild pallor and frequent urination, our patient did not show any other features of adrenal insufficiency. We did not perform a Synacthen test to exclude hypocortisolism due to inadequate funds.

## Conclusion

Patients with HIV infection may develop IRIS as their immunity improves. IRIS may manifest as autoimmune polyglandular syndrome, requiring appropriate care and monitoring to prevent complications due to each APS disease component. The treatment of diabetes mellitus in patients with coexisting hyperthyroidism pose a challenge regarding glucose control because hyperthyroidism induces insulin resistance. Beta blockers in patients with thyrotoxicosis/hyperthyroidism may also put the patient at risk for hypoglycemia unawareness.

## References

[1] Bãnicã D, Frãtilã R, Sima A, Vlad A, Timar (2014). Autoimmune polyglandular syndrome type 2: a case report. Rom J Diabetes. Nutr Metab Dis.

[2] Guo JC, Leung PSC, Zhang W, Ma X, Gershwin ME (2018). The immunobiology and clinical features of type 1 autoimmune polyglandular syndrome (APS-1). Autoimmun Rev [Internet].

[3] Kahaly G J, Fromme L (2018). Polyglandular autoimmune syndromes. J Endocrinol Invest.

[4] Bello MO, Garla VV (2020). Polyglandular Autoimmune Syndrome Type I. StatPearls [Internet].

[5] Sajjadi-Jazi SM, Soltani A, Enayati S, Kakavand Hamidi A, Amoli MM (2019). Autoimmune Polyglandular Syndrome Type 1: A case report. BMC Med Genet.

[6] Beishuizen SJE, Geerlings SE (2009). Immune reconstitution inflammatory syndrome: Immunopathogenesis, risk factors, diagnosis, treatment and prevention. Neth J Med.

[7] Takarabe D, Rokukawa Y, Takahashi Y, Goto A, Takaichi M, Okamoto M (2010). Autoimmune diabetes in HIV-infected patients on highly active antiretroviral therapy. J Clin Endocrinol Metab.

[8] Van den Driessche A, Eenkhoorn V, Van Gaal L, De Block C (2009). Type 1 diabetes and autoimmune polyglandular syndrome: a clinical review. Neth J Med.

[9] Martins SC, Venade G, Teixeira M, Olivério J, Machado J, Marques J (2019). Autoimmune polyglandular syndrome type 2. Revista da Associacao Medica Brasileira.

